# Enantiomeric excess by magnetic circular dichroism in Archaean atmosphere

**DOI:** 10.1038/s41598-017-13653-0

**Published:** 2017-10-16

**Authors:** A. Sharma

**Affiliations:** Department of Physics, Alabama A&M University, Huntsville, AL 35762 USA

## Abstract

Evolution of homochirality requires an initial enantiomeric excess (EE) between right and left-handed biomolecules. We show that magnetic circular dichroism (MCD) of sun’s ultraviolet C light by oxygen in Archaean earth’s anoxic atmosphere followed by chirally selective damage of biomolecules due to circular dichroism (CD) can generate EE of correct handedness. Our calculation of EE uses published data for CD of biomolecules and accepted magnitude for Archaean earth’s magnetic field. Independent of atmospheric oxygen concentration calculated EE has the same sign for all pyrimidine nucleosides which is opposite to that for amino-acids. Purine nucleosides have smaller EE values with opposite sign to pyrimidines but are less susceptible to UV damage. Homochirality is explained by origin of prebiotic life in one hemisphere of earth and its evolution to EE ~ ± 1 before reversal of terrestrial magnetic field. Chirality of biomolecules is decided by the direction of magnetic field where prebiotic life originated on Archaean earth.

## Introduction

While homochirality is pervasive in molecules which form the building blocks of life^1^, results of Urey-Miller experiment^2^ form the basis of hypothesis that life originated on earth with a racemic mixture of prebiotic molecules. It is believed that evolution of homochirality rests on: (i) a mechanism to create initial enantiomeric excess (EE) between right (R) and left (L)-handed biomolecules and (ii) amplification of EE over time to generate 100% homochirality^1,3^. Two possible mechanisms have been proposed to explain the initial EE which evolved to 100% homochirality over a period of time. One of these has an extra-terrestrial source and rests on the observed small EE of L-amino acids found on chondritic meteorites^4^. However, it is possible that some of the evidence on these meteorites may be due to contamination^5^ by the L-amino acids of our biosphere. Second mechanism is stochastic and the observed homochirality is explained as a “by-chance” phenomenon^6,7^. Neither of these two mechanisms can explain why all five nucleosides are R-handed and all twenty standard amino acids (except achiral glycine) have the opposite L-handedness. Presence of terrestrial magnetic field can in principle break the chiral symmetry of biomolecules. In an effect labeled “magnetochiral dichroism”, unpolarized light propagating in the direction of a magnetic field can produce an EE in some chemical reactions involving metal and organic compounds^8^. However, its role in creating an EE for biomolecules like nucleic and amino acids remains to be demonstrated.

We show that initial EE of correct handedness can be generated by MCD of ultraviolet C (UVC) light by paramagnetic oxygen molecules in the atmosphere of Archaean earth followed by chirally selective photo damage due to CD in biomolecules. Dextro (right-handed) ribose and deoxyribose sugar is primarily responsible for the chirality of nucleic acids. These sugars have strong absorption/CD bands only for vacuum UV wavelengths of less than 190 nm^9,10^. As shown in this report, UV flux reaching Archean earth was limited to wavelengths above 200 nm making isolated sugar molecules immune to UV photolysis. Nucleosides are the smallest chiral building blocks of nucleic acids with strong circular dichroism for UV wavelengths in the 200–300 nm range and are analyzed together with amino-acids for EE. The model implies that enantiomeric enrichment by asymmetric photolysis with UV light happened after bigger molecules like nucleosides had formed.

Our analysis utilizes published data for CD of L-amino-acids and R-nucleosides and is based on the accepted understanding of paleomagnetism^11^ and Archaean atmosphere ~4,000 Mya. Evidence from Archaean rocks related to mass independent fractionation of sulphur (MIF-S) isotopes suggests an anoxic atmosphere^12^ with partial pressure of oxygen, p(O_2_) < 10^−2^ PAL (present atmospheric level) although there are reports of loss of MIF-S and possible oxic atmosphere even 3.8 billion years ago^13^. While MIF-S effect still needs to be understood^14^ to make quantitative conclusions, based on photochemical modeling, maximum pressure of atmospheric oxygen^12^ that would support MIF-S is p(O_2_) ~10^−2^ PAL. As shown in this report the sign of EE of nucleosides and amino-acids is independent of oxygen concentration but depends significantly on the pressure of carbon-dioxide in Archaean atmosphere.

## Results

### Transmission of UVC in Archean atmosphere

**Figure 1:**
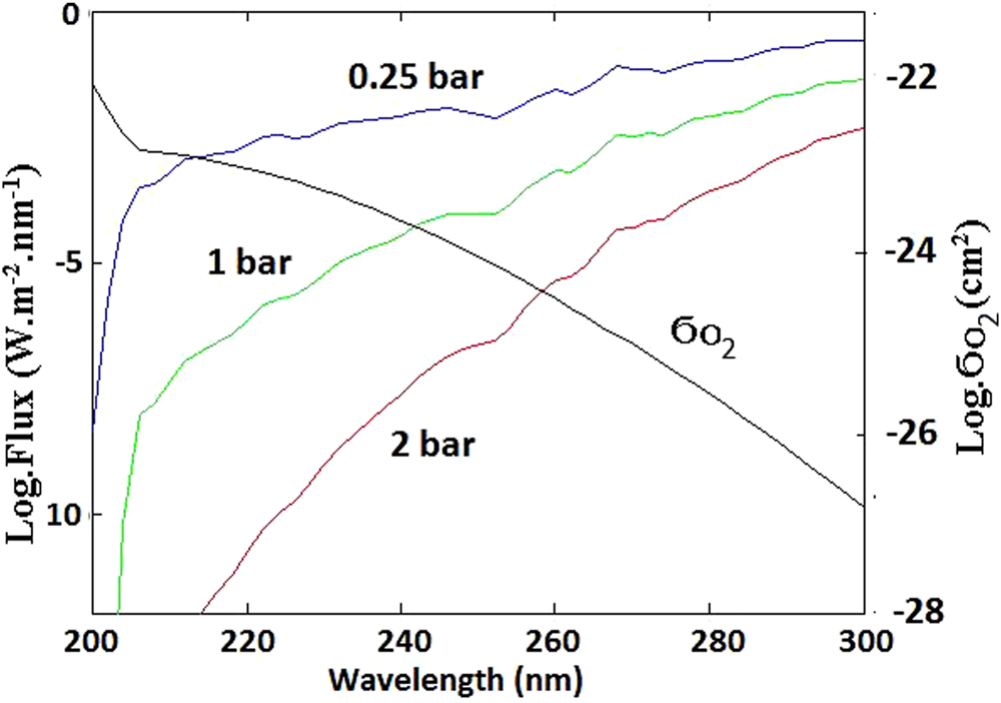
Logarithmic plots of spectral flux of UVC light reaching earth’s surface for atmospheric CO2 pressure of 0.25, 1 and 2 bar and of absorption cross section of molecular oxygen by the forbidden transitions of the Herzberg bands. The figure shows that the spectral region outside of 200–300 nm is unimportant for magnetic circular dichroism by Archean atmospheric oxygen.

For analysis in this report, carbon dioxide and oxygen are the two most important components of Archaean atmosphere when prebiotic life arose around 4 billion years ago. While CO_2_ is primarily responsible for attenuating far UV sunlight in wavelength range of 200–300 nm^15^, O_2_ is the only atmospheric component which is paramagnetic and capable of producing MCD of this UV light. Concentration of O_2_ in atmosphere will determine the extent of MCD of UV light and the differential circular polarization (CP) intensity reaching earth’s surface. Accepted models^16^ for earth’s atmosphere around 4,000 Mya require high CO_2_ pressure (0.1–10 bar) to compensate for lower solar luminosity of young Sun. Figure 1 is a plot of calculated UVC flux reaching early Archaean earth’s surface (p(CO_2_) = 0.25, 1, 2 bar) and shows that wavelengths below 200 nm were strongly attenuated. In plotting these curves, accepted values are used for CO_2_ absorption cross-sections, density-altitude profile and the spectral-intensity of sunlight reaching earth’s upper atmosphere 4,000 Mya^15^. For wavelengths in 200–300 nm, molecular oxygen absorbs weakly by three forbidden transitions^17,18^, giving rise to the Herzberg continuum which originates in paramagnetic X^3^Σ^−^_g_ ground state and is important for atmospheric physics. As seen in Fig. 1, total absorption cross section for these transitions in oxygen (X^3^Σ^−^_g_ → A^3^Σ^+^_u_, X^3^Σ^−^_g_ → c^1^Σ^−^_u_ and X^3^Σ^−^_g_ → A^’3^Δ_u_) rapidly decreases from 200 nm to 300 nm^17–19^. In laboratory, MCD of O_2_ has been observed^20^ within the Herzberg continuum by a matrix-isolation technique at low temperatures and high magnetic field. Attenuation by CO_2_ and absorption cross-section for oxygen in Fig. 1 serves to explain why the effect of MCD by Archaean atmospheric oxygen is unimportant outside the spectral region of 200–300 nm.

### Magnetic circular dichroism of UVC in Archean atmosphere

**Figure 2:**
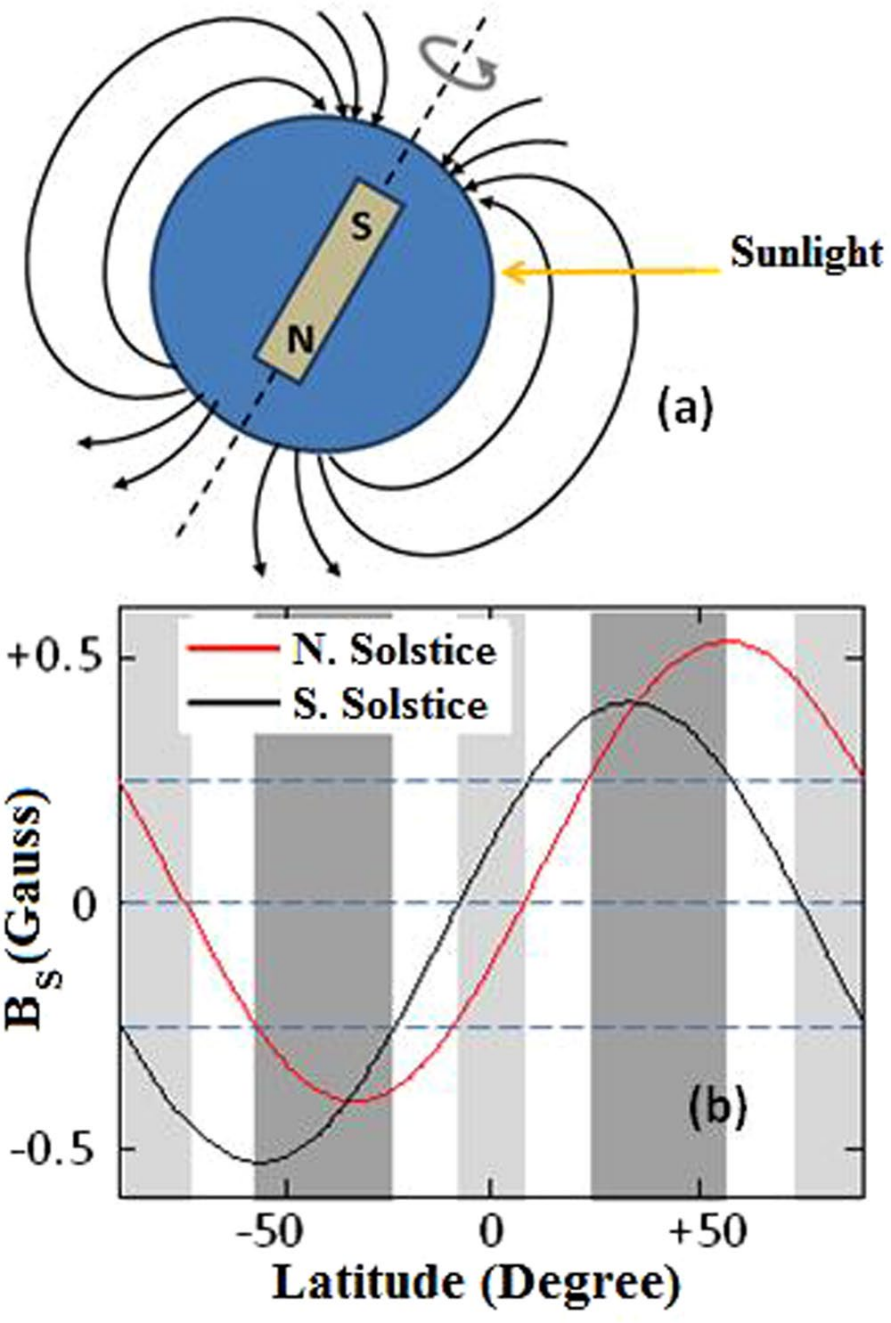
(**a**) Model of terrestrial magnetic dipole for calculating B_S_, the component of field in the direction of sunlight, (**b**). Variation of B_S_ (component of geomagnetic field intensity in the direction of sunlight at zenith) with latitude for the two extremes of solstices. Dipolar magnetic field intensity of 0.3 Gauss at equator is assumed. Dark gray regions of latitude are relatively important for MCD where B_S_ does not change sign throughout the year and |B_S_| > 0.25 Gauss. For light gray regions B_S_ changes sign in the course of a year and so are not important for MCD.

The dipolar geomagnetic field is as old^11^ as the Earth itself with the dipole axis nearly but not exactly aligned with axis of earth’s rotation. Paleomagnetism^11,21^ evidence suggests that the magnitude of magnetic field has not changed significantly since 4,000 Mya. The magnetic poles have wandered by as much as 15–20° but for the purpose of this analysis we will assume that the axis of dipole coincides with the axis of earth’s rotation (Fig. 2). We also assume the magnetic field intensity at the equator of 0.3 Gauss which is the present average value^22^ for this latitude; and the tilt of earth’s axis towards the ecliptic plane as θ_T_ = 23.4° (current value). For MCD, the component (B_S_) of geomagnetic field parallel to the direction of sunlight reaching earth at zenith (midday) varies with the latitude as well as the time of the year and is derived with reference to Fig. 2a. Close to the surface of Earth (radius R), the radial (B_r_) component of the dipolar magnetic field and the component parallel (B_||_) to earth’s surface is given by^22^,

1 $${{\rm{B}}}_{{\rm{r}}}=-2{{\rm{B}}}_{0}\,\mathrm{Sin}({{\rm{\theta }}}_{{\rm{L}}})\quad {\rm{and}}\quad {{\rm{B}}}_{||}={{\rm{B}}}_{0}\,\mathrm{Cos}({{\rm{\theta }}}_{{\rm{L}}})$$

where B_0_ is the magnetic field at the equator and θ_L_ is the latitude of the position on earth. Average value of B_0_ on the equator is 0.3 Gauss.

For the geometry shown in Fig. 2a (northern solstice) when the North Pole is most tilted towards the sun, the component of earth’s magnetic field in the direction of sunlight at zenith (midday) is:

2 $${{\rm{B}}}_{{\rm{S}}}=-{{\rm{B}}}_{{\rm{r}}}\,\mathrm{Cos}({{\rm{\theta }}}_{{\rm{L}}}-{{\rm{\theta }}}_{{\rm{T}}})+{{\rm{B}}}_{||}\,\mathrm{Sin}({{\rm{\theta }}}_{{\rm{L}}}-{{\rm{\theta }}}_{{\rm{T}}})$$

where θ_T_ = 23.4° is the angle of tilt of the earth’s axis towards the ecliptic plane. Similarly, for southern solstice when the North Pole is most tilted away from the sun, the component of earth’s magnetic field in the direction of sunlight at zenith (midday) is:

3 $${{\rm{B}}}_{{\rm{S}}}=-{{\rm{B}}}_{{\rm{r}}}\,\mathrm{Cos}({{\rm{\theta }}}_{{\rm{L}}}+{{\rm{\theta }}}_{{\rm{T}}})+{{\rm{B}}}_{||}\,\mathrm{Sin}({{\rm{\theta }}}_{{\rm{L}}}+{{\rm{\theta }}}_{{\rm{T}}})$$

Both B_r_ and B_||_ vary^22^ with radial distance (r) as (R/r)^3^ which shows that for distances of up to 100 km above earth’s surface B_S_ varies by less than 3%. Atmosphere of this thickness accounts for 99% of all gaseous oxygen around earth^22^.

Magnetic field B_S_ is shown in Fig. 2b for two different times of the year, i.e. for northern and southern solstices. For given latitude, B_S_ varies over period of a year between the two extremes of solstices. As shown by the shaded (dark gray) region in Fig. 2b, only for a small range of latitudes (24°N to 58°N and 24°S to 58°S), B_S_ remains significant (|B_S_| > 0.25 Gauss) throughout the year without changing sign. In the light-gray regions (close to equator and the poles) of Fig. 2b, B_S_ changes sign in the course of a year. With these assumptions, 24°N to 58°N and 24°S to 58°S are the most important geographic regions where the effects of MCD are observed.

**Figure 3:**
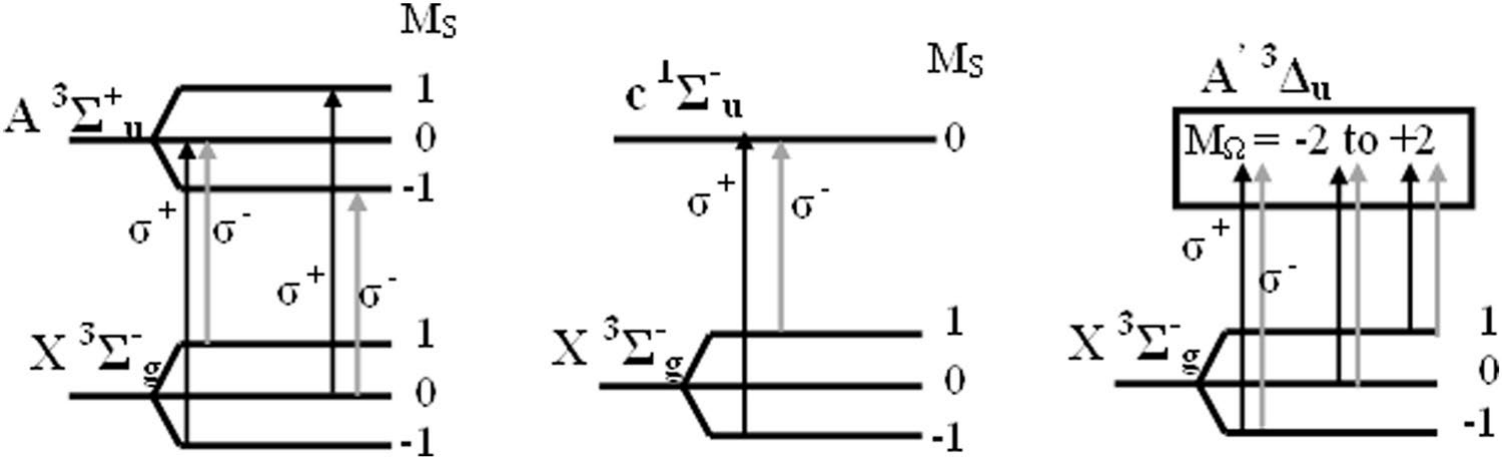
Energy levels for molecular absorption from the X^3^Σ^−^_g_ ground state of paramagnetic molecular oxygen. X → A accounts for bulk of the absorption in the forbidden Herzberg band and is most important for MCD. Due to Boltzmann distribution in the ground state magnetic sublevels, σ^+^ (LCP) light will be absorbed more than σ^−^ (RCP) light and there will be a small relative excess of σ^−^ light reaching earth’s surface for magnetic field in the direction of light propagation.

The significance of MCD-related transitions^20^ in molecular oxygen which results in net circular polarization of UVC light is explained with Fig. 3. Of the three forbidden transitions, X^3^Σ^−^_g_ → A ^3^Σ^+^_u_ is the strongest, accounting for most (86%) of the absorption^18^ in Herzberg continuum. Zeeman splitting of states by the small (~0.25 Gauss) geomagnetic field and possible transitions with left circularly polarized (LCP, σ^+^) and right circularly polarized (RCP, σ^−^) light (propagating in the direction of magnetic field) are shown schematically. In the ground X^3^Σ^−^_g_ state, Boltzmann distribution will result in a small excess of population in M_S_ = −1 over M_S_ = +1. As seen in this figure, for X^3^Σ^−^_g_ → A ^3^Σ^+^_u_ and X^3^Σ^−^_g_ → c ^1^Σ^−^_u_ transitions, M_S_ = −1 in the ground state can only absorb σ^+^ light while M_S_ = +1 can only absorb σ^−^ light. Population difference between M_S_ = −1 and M_S_ = +1 results in slightly greater absorption of σ^+^ light over σ^−^. This is not possible with X^3^Σ^−^_g_ → A^′ 3^Δ_u_ transition (Fig. 3). Net (albeit very small) circular polarization of light reaching earth’s surface is a result of ^3^Σ → Σ transitions in molecular oxygen, i.e. UV light reaching earth will be net σ^−^ when propagating in the direction of earth’s magnetic field and net σ^+^ when propagating against the direction of magnetic field.

Differential (net) circular polarization (CP) intensity due to MCD by atmospheric O_2_ is derived as follows:

If *I*_+_(*λ*) and *I*_−_(*λ*) is the spectral intensity (W.m^−2^ . nm^−1^) of LCP (σ^+^) and RCP (σ^−^) circularly polarized solar UV radiation respectively, reaching the earth’s surface after passing through the atmosphere,

4 $${I}_{+}(\lambda )=\frac{{I}_{0}(\lambda )}{2}{e}^{-{\alpha }_{+}}\quad {\rm{and}}\quad {I}_{-}(\lambda )=\,\frac{{I}_{0}(\lambda )}{2}{e}^{-{\alpha }_{-}}$$

*I*_0_(*λ*) is the initial (above atmosphere) unpolarized intensity (equal components of RCP and LCP). Coefficients *α*_+_ and *α*_*-*_ describe bulk of the atmospheric absorption^23^ by CO_2_ and relatively very weak differential absorption of circularly polarized light (MCD) by atmospheric O_2_.

5a $${\alpha }_{+}(\lambda )=\sigma (C{O}_{2},\,\lambda ).\int N(C{O}_{2},\,L).dL\,+\,{\sigma }_{+}({O}_{2},\,\lambda ).\int {N}_{-}({O}_{2},L).dL$$

Likewise,

5b $${\alpha }_{-}(\lambda )=\sigma (C{O}_{2},\lambda ).\int N(C{O}_{2},L).dL+{\sigma }_{-}({O}_{2},\lambda ).\int {N}_{+}({O}_{2},L).dL$$

The integration is over altitude (L) between 0 and 100 km of atmosphere which adequately^24^ includes absorption by more than 99% of CO_2_ and O_2_.

*σ*(*CO*_2_, *λ*): Absorption cross-section for CO_2_ at wavelength λ

*N*(*CO*_2_, *L*): Number density of CO_2_ at altitude L

*σ*_+_(*O*_2_, *λ*): absorption cross-section for O_2_ at wavelength λ (LCP light)

*σ*_*−*_(*O*_2_, *λ*): absorption cross-section for O_2_ at wavelength λ (RCP light)

*N*_*−*_(*O*_2_, *L*): Number density of O_2_ in M_S_ = −1 at altitude L

*N*_+_(*O*_2_, *L*): Number density of O_2_ in M_S_ = +1 at altitude L

For small magnetic field, H ≈ 0.25 Gauss, the magnetic sublevels of O_2_ are degenerate and we assume, *σ*_+_(*O*_2_, *λ*) = *σ*_*−*_(*O*_2_, *λ*) ≡ *σ*(*O*_2_, *λ*)

Boltzmann distribution gives,

6 $$(\frac{{N}_{-}({O}_{2},L)}{{N}_{+}({O}_{2},L)})={e}^{{\rm{\Delta }}E(H)/kT}$$

Here, *ΔE*(*H*) = *µ*_*B*_ . *g* . *ΔM*_*S*_.*H* is the energy difference between M_S_ = −1 and M_S_ = 1 Zeeman levels for magnetic field *H*; *µ*_*B*_ is the Bohr magneton, *g* is the Lande factor, *ΔM*_*S*_ = *2* for the two relevant sublevels of the ground state X^3^Σ^−^_g_ and *k* is the Boltzmann constant. We assume, *H* ≡ *B*_*S*_ = 0.25 Gauss, Lande *g* factor^25^ ≈2 and *T* = 300 K giving, *ΔE/kT* = *2*.*24* × *10* ^−7^.

Due to three nearly degenerate magnetic sublevels of the ground state of O_2_,

7 $${N}_{-}({O}_{2},L)=\frac{N({O}_{2},L)}{3}\quad {\rm{and}}\quad {N}_{+}({O}_{2},L)=\frac{N({O}_{2},L)}{3}.\,{e}^{-{\rm{\Delta }}E(H)/kT}$$

**Figure 4:**
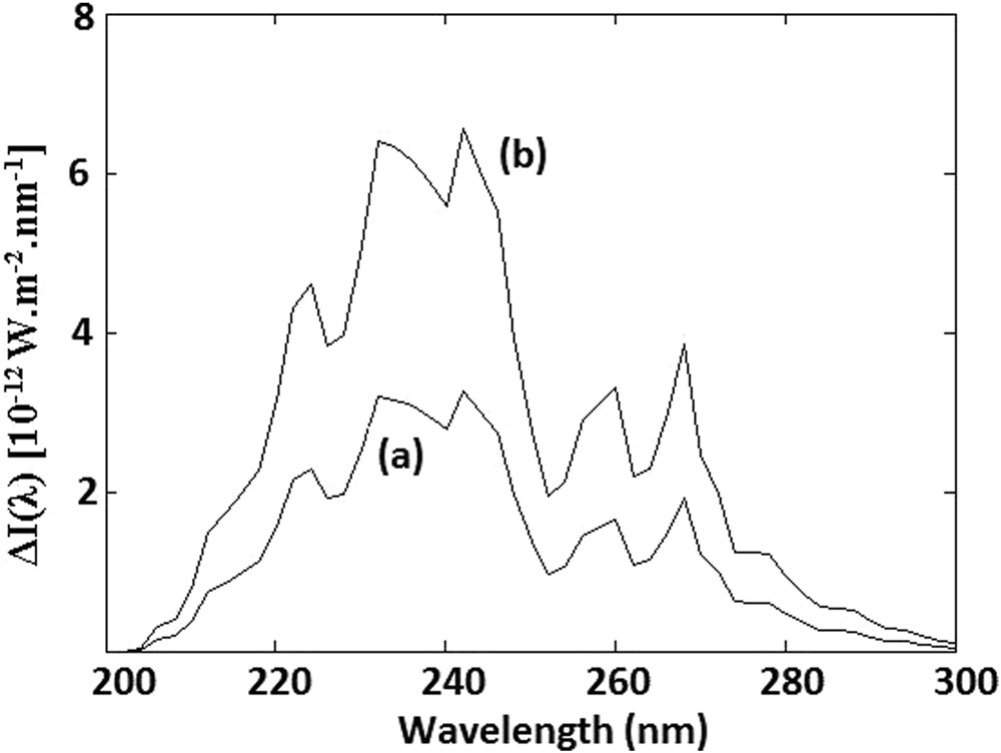
Differential circular polarization intensity for p(O_2_) = 0.002 bar and p(CO_2_) = 0.5 bar. (**a**) ΔE/kT = 2.24 × 10^−7^, (**b**) ΔE/kT = 4.48 × 10^−7^.

*N*(*O*_2_, *L*) is the total number density of O_2_ at altitude *L*. Spectral intensities *I*_+_(*λ*) and *I*_*−*_(*λ*) are calculated from above equations, published data for absorption cross-sections *σ*(*CO*_2_, *λ*)^15^ and *σ*(*O*_2_, *λ*)^17–19^, and the density-altitude profile^15^ for given surface pressure of CO_2_. We assume complete mixing of CO_2_ and O_2_, i.e., *N*(*O*_2_, *L*)/*N*(*CO*_2_, *L*) is independent of altitude *L*. Differential circular polarization (CP) intensity *ΔI*(*λ*) ≡ (*I*_−_(*λ*) − *I*_+_(*λ*)) is calculated from Eqns 4–7 and shown in Fig. 4 for a surface O_2_ pressure p(O_2_) = 10^−2^ PAL = 0.002 bar and CO_2_ pressure of 0.5 bar. Similar plots can be generated for any other surface O_2_ and CO_2_ pressure. As seen in this figure, *ΔI*(*λ*) is proportional to *B*_*S*_/*T*.

### Enantiomeric excess for biomolecules

Calculation of EE is made for biomolecules in aqueous medium as is the case with most theories for origin of prebiotic life in marine/freshwater environment^26,27^. The products of Miller Urey type reactions with atmospheric gases collected over the large surface area of Archean oceans. Concentration of amino acids in Archean oceans is estimated to vary^27,28^ from 4 × 10^−3^ M to 10^−7^ M. Concentration of five nucleobases in one Miller-Urey experiment^29^ was measured to be between 1–100 ppm. While these numbers are small, there are several proposed mechanisms for amplifying the concentration of prebiotic molecules on ocean shorelines and drying freshwater ponds^30^. Additionally, enantiomeric excess depends on the ratio and not absolute values of enantiomeric concentrations. As shown in a recent report^31^, around 4000 Mya the pH of seawater was in the 6.5–7.0 range and not too different from freshwater. Data used in this report is for solutions of pH around 7. While asymmetric photolysis and enantiomeric enrichment of amino acids by circularly polarized light in liquid varies with the acidic pH in the range of 2–6, there is no effect of pH in the 6.5–7.0 range or higher^32,33^.

Chiral molecules like nucleic acid monomers and amino acids show circular dichroism^34,35^ in the 200–300 nm region of UV light. Starting with a racemic population of these prebiotic building blocks of life, exposure to net circularly polarized UVC light will result in an enantiomeric excess that will be a seed for evolution to homochirality. Such an explanation has been proposed^4^ for the observed enantiomeric excess of L-amino acids in chondritic meteorites and assumes that the UV damage/inactivation rates D_R_, D_L_ of right-/left-handed biomolecules are proportional to light absorption rates. This is also seen from the observed similarity of absorption spectrum and action spectrum for direct photo-damage of nucleic acids by UVC radiation^36,37^. Using published CD parameter Δε(λ)^34,35^, extinction coefficient ε(λ)^35,38–40^ and calculated differential CP intensity ΔI(λ) ≡ (I_-_(λ) − I_+_(λ)), the differential UV damage rate ΔD/D ≡ (D_R_ − D_L_)/ (D_R_ + D_L_) for nucleosides and amino acids is derived below. As shown here, enantiomeric excess is related to ΔD/D.

Cross sections for absorption of UV by biomolecules (amino-acids/nucleosides) are listed below.

*σ*_*R*_^+^(*λ*): Cross-section for absorption of σ^+^ light by right-handed (RH) molecule

*σ*_*R*_^−^(*λ*): Cross-section for absorption of σ^−^ light by right-handed (RH) molecule

*σ*_*L*_^+^(*λ*): Cross-section for absorption of σ^+^ light by left-handed (LH) molecule

*σ*_*L*_^−^(*λ*): Cross-section for absorption of σ^−^ light by left-handed (LH) molecule

For the two kinds of chiral molecules (RH/LH) and two circular light polarizations (σ^+^ and σ^−^), following rate equations are valid. LCP (σ_+_) energy absorbed per sec by one RH molecule is^41^

8a $$\,{\Gamma }_{R}^{+}=\,{\int }^{}{I}_{+}(\lambda ).{\sigma }_{R}^{+}(\lambda ).d\lambda \,$$

Similarly, absorption rates for other three combinations,

8b $$\,{\Gamma }_{R}^{-}=\,{\int }^{}{I}_{-}(\lambda ).{\sigma }_{R}^{-}(\lambda ).d\lambda \,$$

8c $$\,{\Gamma }_{L}^{+}=\,{\int }^{}{I}_{+}(\lambda ).{\sigma }_{L}^{+}(\lambda ).d\lambda \,$$

8d $$\,{\Gamma }_{L}^{-}=\,{\int }^{}{I}_{-}(\lambda ).{\sigma }_{L}^{-}(\lambda ).d\lambda \,$$

Since chiral molecules can absorb both σ^+^ and σ^−^ components of UV light, total absorption rates for RH and LH enantiomers are:

9a $${\Gamma }_{R}=\,{\Gamma }_{R}^{+}+\,{\Gamma }_{R}^{-}$$

9b $${\Gamma }_{L}=\,{\Gamma }_{L}^{+}+\,{\Gamma }_{L}^{-}$$

From chiral symmetry, σ_R_^+^(λ) = σ_L_^−^(λ) and σ_R_^−^(λ) = σ_L_^+^(λ). As before, differential circular polarization (CP) intensity due to MCD by atmospheric O_2_ is *ΔI*(*λ*) ≡ *I*_−_(*λ*) − *I*_+_(*λ*). Define, *Δσ*(*λ*) ≡ *σ*_*L*_^+^(*λ*) − *σ*_*L*_^−^(*λ*) = *σ*_*R*_^−^(*λ*) − *σ*_*R*_^+^(*λ*). It follows from above rate Eqs 8 and 9,

10a $$\,{\Gamma }_{R}-{\Gamma }_{L}\,=\,\int {\rm{\Delta }}I(\lambda ).{\rm{\Delta }}\sigma (\lambda ).d\lambda \,$$

Similarly,

10b $$\,{\Gamma }_{R}+{\Gamma }_{L}\,=\,{\int }^{}{I}_{0}(\lambda ).\sigma (\lambda ).d\lambda \,$$

Here, *I*_−_(*λ*) ≈ *I*_+_(*λ*) = *I*_0_(*λ*)/*2* and *σ*(*λ*) = *σ*_*L*_^+^(*λ*) + *σ*_*L*_^−^(*λ*) = *σ*_*R*_^+^(*λ*) + *σ*_*R*_^−^(*λ*).

Molar circular dichroism is defined as *Δε* = *ε* ^+^ − *ε* ^−^, where *ε* ^+^ and *ε* ^−^ are the molar extinction coefficients (mol^−1^ . cm^−1^) for σ^+^ and σ^−^ light respectively. Absorption cross-section *σ*(*λ*) is proportional^42^ to *ε*(*λ*). Thus,

11a $$\frac{{\Gamma }_{R}-{\Gamma }_{L}\,}{{\Gamma }_{R}+{\Gamma }_{L}}=\frac{\int {\rm{\Delta }}I(\lambda ).{\rm{\Delta }}\sigma (\lambda ).d\lambda \,}{\int {I}_{0}(\lambda ).\sigma (\lambda ).d\lambda \,}=\frac{\int {\rm{\Delta }}I(\lambda ).{\rm{\Delta }}{\varepsilon }_{L}(\lambda ).d\lambda \,}{\int {I}_{0}(\lambda ).\varepsilon (\lambda ).d\lambda \,}=-\frac{\int {\rm{\Delta }}I(\lambda ).{\rm{\Delta }}{\varepsilon }_{R}(\lambda ).d\lambda \,}{\int {I}_{0}(\lambda ).\varepsilon (\lambda ).d\lambda \,}$$

Here, *Δε*_*L*_ = *ε*_*L*_^+^ − *ε*_*L*_^−^ for L-handed molecule and *Δε*_*R*_ = *ε*_*R*_^+^ − *ε*_*R*_^−^ for R-handed molecule. Since *Δε*(*λ*) is typically less than 1% of *ε*(*λ*), in the denominator *ε*(*λ*) ≈ *ε*_*L*_(*λ*) ≈ *ε*_*R*_(*λ*). It is commonly assumed that the UV destruction/damage rate (D) for nucleic acids^36,37^ is proportional to the light energy absorption rate (Γ). Thus,

11b $$\frac{{\rm{\Delta }}D\,}{D}\equiv \frac{{D}_{R}-{D}_{L}\,}{{D}_{R}+{D}_{L}}=\frac{{\int }^{}{\rm{\Delta }}I(\lambda ).{\rm{\Delta }}{\varepsilon }_{L}(\lambda ).d\lambda \,}{{\int }^{}{I}_{0}(\lambda ).\varepsilon (\lambda ).d\lambda \,}=-\frac{\int {\rm{\Delta }}I(\lambda ).{\rm{\Delta }}{\varepsilon }_{R}(\lambda ).d\lambda \,}{\int {I}_{0}(\lambda ).\varepsilon (\lambda ).d\lambda \,}$$

Relative efficiency for photolysis has been studied only for a few amino acids and for some isolated wavelengths. As an example, photolysis of phenylalanine at 206 nm and 254 nm does not show any significant effect of wavelength^43^. Further, CD of amino acids is significant only over a band-width of 20–30 nm^34^. Above equation to calculate ΔD/D is used for nucleosides and amino acids.

In eqn. 11, Δε_L_(λ), Δε_R_(λ) is the molar CD parameter for L/R-handed enantiomer. Enantiomeric excess (N_R_ − N_L_)/(N_R_ + N_L_) is the relative excess of right-handed molecular concentration (N_R_) over left-handed (N_L_) molecules. It is related to ΔD/D and the sign of EE is opposite to that of ΔD/D (if D_R_ > D_L_, N_R_ < N_L_). In a simple model described below, for racemic production and UV damage of biomolecules enantiomeric excess, EE = −(ΔD/D).

### Model for racemic production and UV damage of biomolecules

Correlation between EE and the parameter (ΔD/D) is explained with the help of a simple phenomenological model that describes the racemic production of prebiotic life-molecules and their destruction by Archaean UVC radiation. For number density N_R_ and N_L_ of right and left handed molecules,

12 $$\frac{d{N}_{R}}{dt}=-{D}_{R}{N}_{R}+P\quad {\rm{and}}\quad \frac{d{N}_{L}}{dt}=-{D}_{L}{N}_{L}+P$$

The terms −D_R_N_R,_ −D_L_N_L_ describe destruction by UV light with rate constants D_R_ and D_L_ which could be different due to differential circular polarization intensity, *ΔI*(*λ*) ≡ *I*_*−*_(*λ*) − *I*_+_(*λ*). The racemic production rate P is same for both enantiomers. For time, t ≫ D_R_^−1^, D_L_^−1^ the steady-state distribution (dN_R,L_/dt ≈ 0) results in an enantiomeric excess,

13a $$EE\equiv \frac{{N}_{R}-{N}_{L}\,}{{N}_{R}+{N}_{L}}=-(\frac{{D}_{R}-{D}_{L}\,}{{D}_{R}+{D}_{L}})\equiv -\frac{{\rm{\Delta }}D\,}{D}$$

For time, t ≪ D_R_^−1^, D_L_^−1^

13b $$EE\equiv \frac{{N}_{R}-{N}_{L}\,}{{N}_{R}+{N}_{L}}=-({D}_{R}-{D}_{L}).\frac{t}{4}\equiv -\frac{{\rm{\Delta }}D\,}{D}(\frac{Dt}{4})$$

Equation 11b is used to calculate EE (−ΔD/D) using published values of CD parameter *Δε*(*λ*)^34,35^ and extinction coefficient *ε*(*λ*)^35,38–40^ for R-nucleosides and L-amino-acids. A summary of reported^34,35^ CD bands for these biomolecules is given in Table 1.

**Table 1:** Summary of Circular Dichroism (CD) bands for L-amino-acids and R-nucleosides.

L-amino-acids^34^: All CD bands are positive for wavelength in 200–300 nm range	
Alanine	Peak wavelength: 203 nm; Bandwidth (FWHM): 22 nm
Serine	Peak wavelength: 205 nm; Bandwidth (FWHM): 25 nm
Lysine	Peak wavelength: 212 nm; Bandwidth (FWHM): 23 nm
Proline	Peak wavelength: 210 nm; Bandwidth (FWHM): 19 nm
Phenylalanine	Peak wavelength: 219 nm; Bandwidth (FWHM): 9 nm
**R-nucleosides** ^35^ **(pyrimidines)**	
Cytidine	Negative band between 200–240 nm; positive bands for 240–300 nm
Thymidine	Negative band between 208–254 nm; positive bands for 254–300 nm and 180–208 nm
Uridine	Negative band between 208–250 nm; positive bands for 250–290 nm and 182–208 nm
**R-nucleosides** ^35^ **(purines)**	
Adenosine	Positive band for 210–240 nm; negative bands for 240–280 nm and 170–210 nm
Guanosine	Positive band for 206–232 nm; negative bands for 232–290 nm and 170–206 nm

Calculated values of EE for different pressures of atmospheric CO_2_ and O_2_ is given in Table 2 for nucleosides and five randomly selected amino-acids for a scenario where the intensity of σ^−^ light reaching earth’s surface is more than σ^+^ intensity (*ΔI*(*λ*) ≡ *I*_−_(*λ*) − *I*_+_(*λ*) > 0).

## Discussion

UV damage rates D_R,L_ depend on the action spectrum weighted, integrated UVC flux. For 200–300 nm and CO_2_ pressure of 1 bar, total integrated UVC flux is 0.63 W/m^2^ (Fig. 1) and is equivalent to 0.21 W/m^2^ when weighted with the DNA action spectrum^37^. Several studies have measured wavelength specific damage rate constants for nucleic acids which is largely due to photo-damage of pyrimidine bases. From the measured nucleic acid damage rate constant of 0.061 cm^2^/mJ for wavelength of 260 nm^44^, D_R,L_ has a value of 1.2 × 10^−3^ s^−1^. When subjected to Archean UVC flux, pyrimidine nucleosides will be damaged in a time of few minutes. The UV damage rate for purine nucleosides is much smaller than for pyrimidine nucleosides (D_purine_ ≈ 0.01 D_pyrimidine_)^45^ and the corresponding damage time for purine nucleosides in the Archaean UV flux will be several hours. As seen from Equation 13 a,b, for the estimated Archaean UVC flux, EE for pyrimidines will saturate to −ΔD/D in a time of few minutes but will take several hours for purines to do so. Compared to pyrimidines, the Archaean purine population density is racemic (EE ≈ 0).

Relatively fewer studies have measured wavelength specific UV photolysis rate constants for amino acids. Photolysis of amino acids for wavelengths shorter than 200 nm was investigated for space environment^46,47^ outside of terrestrial atmosphere where UV light at these wavelengths is relatively abundant. Photolysis has also been investigated for wavelengths longer than 200 nm^48^ and these are the only ones relevant to our investigation due to absence of vacuum UV light on Archean earth. In one investigation^43^, a comparison was made of the relative effectiveness for amino-acid photolysis at wavelengths of 147 nm, 206 nm and 254 nm. The results show that photolysis effectiveness at these three wavelengths is comparable. For UV flux of 2.66 W/m^2^ at a wavelength of 254 nm, the measured half-life of phenylalanine was 130 min and for UV flux of 8.34 W/m^2^ at a wavelength of 206 nm, it was 38 min. Without any wavelength specific damage action spectra for amino acids for 200–300 nm, it is not possible to correlate these numbers with the Archaen UVC flux shown in Fig. 1. However, in the absence of any dramatic variation of photolysis rates with UVC wavelength, amino-acid half-life of a few hours is expected^43^.

The UV absorption coefficient of water varies from 7 m^−1^ for a wavelength of 200 nm to 0.7 m^−1^ for 300 nm and has a value of 1.7 m^−1^ for the central wavelength of 250 nm^49^. Thus UV light in this wavelength range easily penetrates around 0.5 meters of water. The products of Urey Miller type reactions will continue interacting with UV light till the molecules have sunk to depths greater than ~0.5 meters by diffusion. Diffusion coefficient (D) for molecules of the size of nucleosides and amino acids in water has a value^50^ of ~1.5 × 10^−5^ cm^2^/s. Time to diffuse^51^ down by a distance (L) of 0.5 m is (T ≈ L^2^/4D) several months. This can be compared to a time of several minutes to a few hours required for photodamage of biomolecules in the Archean UV light flux, as explained above. Thus biomolecules in water get adequately illuminated for the photodamage processes described in this report with near-full UV flux from sun.

As seen from Table 1, Δε_L_ is positive^34^ for CD of L-amino-acids in 200–300 nm wavelength region. From equation 11b, ΔD/D is positive for ΔI(λ) > 0. Thus EE is negative (N_L_ > N_R_) for amino-acids and the sign of EE is independent of CO_2_ pressure (Table 2). Unlike amino-acids, the CD spectra of nucleosides show both positive and negative bands^35^ for Δε_R_ (Table 1). Increasing atmospheric CO_2_ shifts the UV pass-band (Fig. 1) and the differential circular polarization (Fig. 4) to longer wavelengths, resulting in a change of sign of ΔD/D (and EE) for pyrimidine nucleosides. As seen in Table 2, for pyrimidine nucleosides EE is positive (N_R_ > N_L_) above a threshold CO_2_ pressure of around 1 bar. In these calculations, the pressure of atmospheric O_2_ is kept at 0.002 bar (0.01 PAL). At low pressures of O_2_, calculated values of EE is approximately proportional to O_2_ pressure. This is shown (Table 2) for CO_2_ pressure of 1.25 bar and lower O_2_ pressure of 0.0002 bar. While magnitude of EE values reduce by a factor of 10, the sign of EE remains unchanged.

**Table 2:** Calculated Enantiomeric Excess (EE) for atmospheric CO_2_ pressure between 0.25–2 bar and an O_2_ pressure of 0.002 bar. EE values are for *I*_−_(*λ*) > *I*_+_(*λ*), i.e. ^*Δ*^ *ΔI*(*λ*) > *0* and when UVC light reaching earth’s surface is preferentially *σ* ^−^ (RCP).

**P(CO**_**2**_ **) bar**	**P(O**_**2**_ **) Bar**	**Enantiomeric Excess (−ΔD/D)**									
		Amino Acids (EE in unit of 10^−10^)					Nucleosides (EE in unit of 10^−15^)				
							Pyrimidines			Purines	
		Ala	Ser	Lys	Pro	Phe	Cytidine	Thymidine	Uridine	Adenosine	Guanosine
0.25	0.002	−0.81	−1.2	−1.3	−0.69	−0.0088	−29.1	−33.7	−35.6	2.2	10.7
0.50	0.002	−0.77	−1.2	−1.5	−0.78	−0.0041	−0.11	−8.0	−5.5	−1.2	1.2
0.75	0.002	−0.77	−1.2	−1.7	−0.87	−0.0013	4.5	−1.0	2.4	−1.7	−0.23
1.00	0.002	−0.81	−1.3	−2.0	−0.97	−0.00039	4.5	0.93	4.4	−1.5	−0.29
1.25	0.002	−0.91	−1.5	−2.4	−1.1	−0.00012	3.8	1.5	4.6	−1.2	−0.21
1.25	0.0002	−0.085	−0.014	−0.22	−0.010	−0.000012	0.38	0.14	0.46	−0.12	−0.021
1.50	0.002	−1.1	−1.7	−2.9	−1.3	−0.000039	3.2	1.5	4.3	−0.94	−0.13
1.75	0.002	−1.4	−2.0	−3.7	−1.5	−0.000013	2.6	1.4	3.9	−0.72	−0.088
2.00	0.002	−1.8	−2.5	−4.8	−1.8	−0.0000046	2.2	1.3	3.4	−0.55	−0.060

Interestingly, the magnitude of EE for both purine nucleosides is smaller and the sign is opposite to that for pyrimidines. However, as explained above (Eqn. 13), compared to pyrimidines EE will take much longer time to reach the saturation value of −ΔD/D and the population of purines is relatively racemic. Further, heterochiral nucleic acids involving pyrimidine and purine building blocks are significantly more unstable thermally^52,53^ as compared to homochiral counterparts. It is conceivable that smaller EE values for purines and significant thermal stability of homochiral nucleic acids together with D_purine_ ≈ 0.01(D_pyrimidine_)^45^ was a determining factor (not the sign of their enantiomeric excess) in the evolution of chirality of purines. Values of ΔD/D for aromatic amino-acids are lower by a factor of ~100 due to larger extinction coefficient ε(λ)^38^ and relatively small circular dichroism Δε(λ)^34^. In all of these calculations we assume an ambient temperature (T) of 300 K and a magnetic field (B_S_) of 0.25 Gauss. Varying conditions of atmospheric temperature and magnetic field can be accounted by the observation that differential circular polarization intensity ΔI(λ) and EE are proportional to B_S_/T (Fig. 4).

## Conclusions

We have shown that magnetic circular dichroism of UVC by sparse oxygen in the anoxic Archaean atmosphere results in net circularly polarized light reaching earth’s surface. This results in chirally selective damage of prebiotic molecules by circular dichroism and creates an EE which evolved to homochirality of R-nucleosides and L-amino-acids. Irrespective of the partial pressure of oxygen, the sign of enantiomeric excess of pyrimidine nucleosides and the amino-acids is predicted correctly when UVC light reaching earth has net σ^−^ circular polarization (*ΔI*(*λ*) ≡ *I*_−_(*λ*) − *I*_+_(*λ*) > *0*) and P(CO_2_) > 1 bar. This is consistent with the requirement of large CO_2_ pressure for greenhouse effect^16^ to balance lower solar luminosity 4,000 Mya. It is also an indication that initial enantiomeric excess was generated by circularly polarized UV light and not by a stochastic event.

Since MCD depends on the direction of magnetic field with respect to the propagation of light, net σ^−^ light in one hemisphere of earth would also result in net σ^+^ light in the other hemisphere. Homochirality in life-molecules can be explained by assuming (i) prebiotic life originated either in the northern or the southern hemisphere of earth which had higher UVC flux of σ^−^ over σ^+^ (south magnetic hemisphere), (ii) evolution of homochirality was complete (EE ~ ±1) before the earth’s magnetic field reversed. Recorded length of polarity intervals between reversals varies between 0.1 and several Million years^54^ and (iii) no significant dispersal of prebiotic life molecules happened between hemispheres before the transition to homochirality was complete. For the terrestrial magnetic field in Fig. 2, the most important regions for effective MCD in atmospheric oxygen lie between latitudes of 24° and 58° in both hemispheres and any EE will be nullified by large-scale dispersal of enantiomers between these regions from south to north magnetic hemisphere.

## Methods

### Calculation of spectral flux I(λ) (W . m^−2^ . nm^−1^) of UVC light reaching earth’s surface Calculation of differential circular polarization intensity ΔI(λ) (W.m^−2^.nm^−1^) of UVC light reaching earth’s surface

Equations 4 and 5 together with published data^15^ for the following is used:

1a. Spectral flux *I*_*0*_(*λ*) (200–300 nm) from model young sun (~4,000 Mya) reaching earth (before entering the atmosphere)

1b. Absorption cross-sections (200–300 nm) for CO_2_

1c. Variation of density with altitude (up to 120 km) for CO_2_ and O_2_. Altitude up 100 km accounts for 99% of these gases.

1d. Absorption cross-sections (200–300 nm) for O_2_ is from refs^17–19^.

### Calculation of enantiomeric excess (−ΔD/D) from equation 11b

Published data (200–300 nm) for the following is used:

2a. Extinction coefficient ε (mol−1 . cm−1) for the four amino acids (Ala, Ser, Lys, Pro) is significant^38^ only for 200–230 nm. For aromatic amino acid (Phe), ε is significant between 200–270 nm.

2b. Circular dichroism (milli.deg) for amino acids^34^ is converted to Δε (mol−1 . cm−1) and is significant only for 200–240 nm

Due to a small range of wavelengths for amino-acids, published data for Δε and ε values is taken in steps of 1 nm.

Following equation (11b) is used to evaluate EE (−ΔD/D) for amino-acids

$$EE=-\frac{{\rm{\Delta }}D\,}{D}=-\frac{\int {\rm{\Delta }}I(\lambda ).{\rm{\Delta }}{\varepsilon }_{L}(\lambda ).d\lambda \,}{{\int }^{}{I}_{0}(\lambda ).\varepsilon (\lambda ).d\lambda \,}=-\frac{{\sum }_{\lambda =200nm}^{240}{\rm{\Delta }}I(\lambda )\,.\,{\rm{\Delta }}{\varepsilon }_{L}(\lambda )}{{\sum }_{\lambda =200nm}^{230,\,270}I(\lambda ).{\varepsilon }_{L}(\lambda )}$$

Summation is done in steps of 1 nm.

2c. Extinction coefficient^39,40^ ε (mol^−1^ . cm^−1^) and circular dichroism Δε (mol^−1^ . cm^−1^) for the five nucleosides^35^ in 200–300 nm

Due to a larger range of wavelengths for nucleosides, published data for Δε and ε values is taken in steps of 2 nm.

Following equation (11b) is used to evaluate EE (−ΔD/D) for five nucleosides

$$EE=-\frac{D\,}{D}=\frac{\int {\rm{\Delta }}I(\lambda ).{\rm{\Delta }}{\varepsilon }_{R}(\lambda ).d\lambda \,}{\int {I}_{0}(\lambda ).\varepsilon (\lambda ).d\lambda \,}=\frac{{\sum }_{\lambda =200nm}^{300}{\rm{\Delta }}I(\lambda ).{\rm{\Delta }}{\varepsilon }_{R}(\lambda )}{{\sum }_{\lambda =200nm}^{300}I(\lambda ).{\varepsilon }_{R}(\lambda )}$$

Summation is done in steps of 2 nm. As seen from Figs 1 and 4, there is very little UV light intensity for wavelengths shorter than 220 nm and there is insignificant contribution to EE from this wavelength region. Likewise, differential circular polarization intensity (Fig. 4) is minimal for wavelength longer than 290 nm and again there is insignificant contribution from this long-wavelength region.

### Data availability

The author declares that the main data supporting the findings of this study are available within this article. Additional data used in this study is available publicly in published journal articles. Appropriate references are provided (Methods section) for the sources of this data.
